# Supplementary material for: Ubiquitylation of nucleic acids by DELTEX ubiquitin E3 ligase DTX3L
Source: EMBO Rep. 2024 Sep 6;25(10):11. doi: 10.1038/s44319-024-00235-1 (PMC11467253; doi:10.1038/s44319-024-00235-1)
Supplement: Supplementary file 1 — Appendix [file 44319_2024_235_MOESM1_ESM.pdf]

Appendix

Appendix Figures S1-9

Table of Contents

Appendix Figure S1. Chemical structures of AMP and ADPr. 2

Appendix Figure S2. HPLC-MS analysis of the ubiquitylation mixture performed using DTX3L-RD and AMP. 3

Appendix Figure S3. HPLC-MS analysis of the ubiquitylation mixture performed using DTX3L-RD and ADPr. 4

Appendix Figure S4. HPLC-MS analysis of the ubiquitylation mixture performed using DTX3L-RD and 2' deoxy-ATP. 5

Appendix Figure S5. HPLC-MS analysis of the ubiquitylation mixture performed using DTX3L-RD and 3' deoxy-ATP. 6

Appendix Figure S6. HPLC-MS analysis of the ubiquitylation mixture performed using DTX3L-RD and GMP. 7

Appendix Figure S7. HPLC-MS analysis of the ubiquitylation mixture performed using DTX3L-RD and CMP. 8

Appendix Figure S8. HPLC-MS analysis of the ubiquitylation mixture performed using DTX3L-RD and TMP. 9

Appendix Figure S9. Differences between WWE domain-containing DELTEXes and KH domain-containing DELTEXes. 10

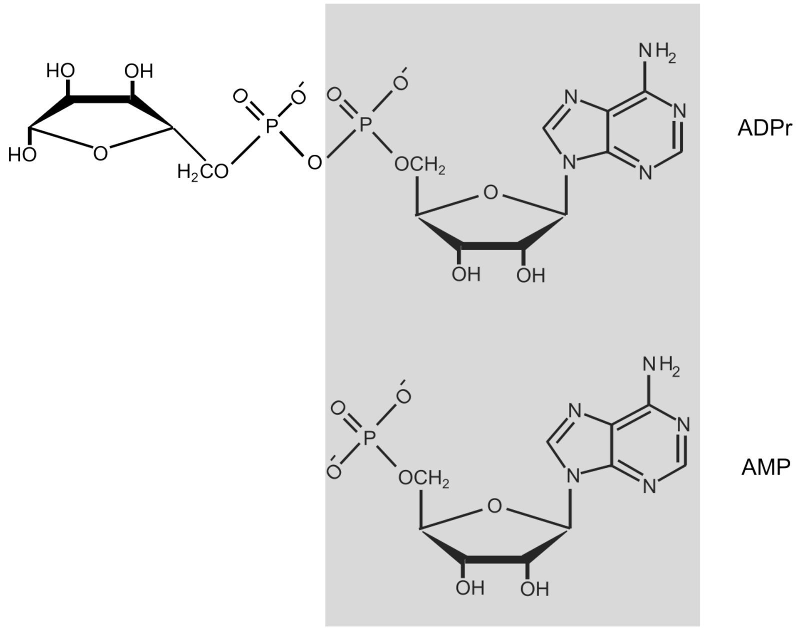

Appendix Figure S1. Chemical structures of AMP and ADPr.

ADPr contains AMP core in its structure.

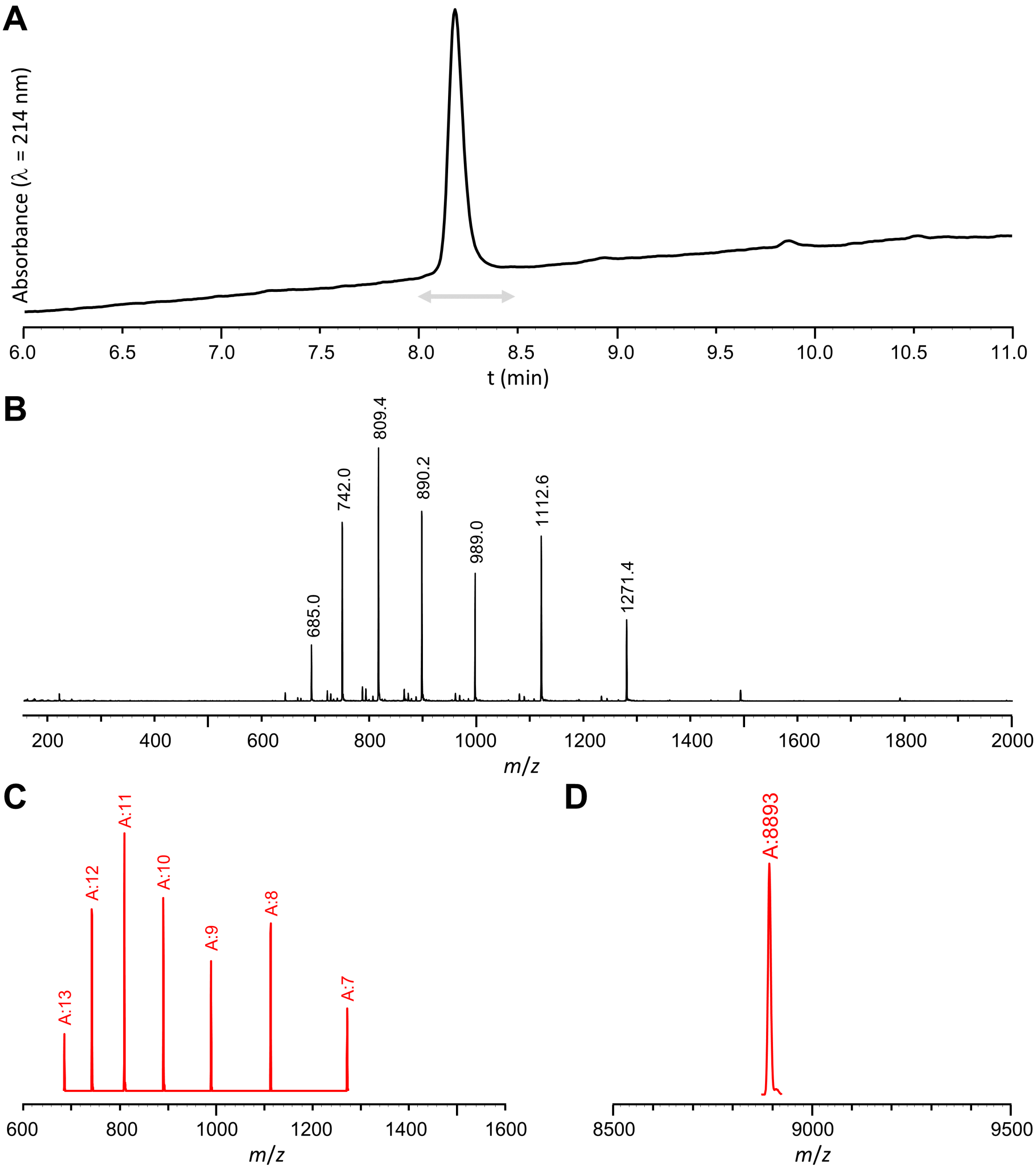

E

Compound	Relative abundance (%)	Detected average mass	Identified product, its theoretical average mass
A	100	8892.7 Da	Ub-AMP, 8893.9 Da

Appendix Figure S2. HPLC-MS analysis of the ubiquitylation mixture performed using DTX3L-RD and AMP.

(A) HPLC chromatogram;

(B) Experimental mass spectrum corresponding to the time window indicated as a grey arrow (sum of spectra);

(C) Deconvoluted ions set, including charge state;

(D) Deconvoluted spectrum;

(E) Identified compounds.

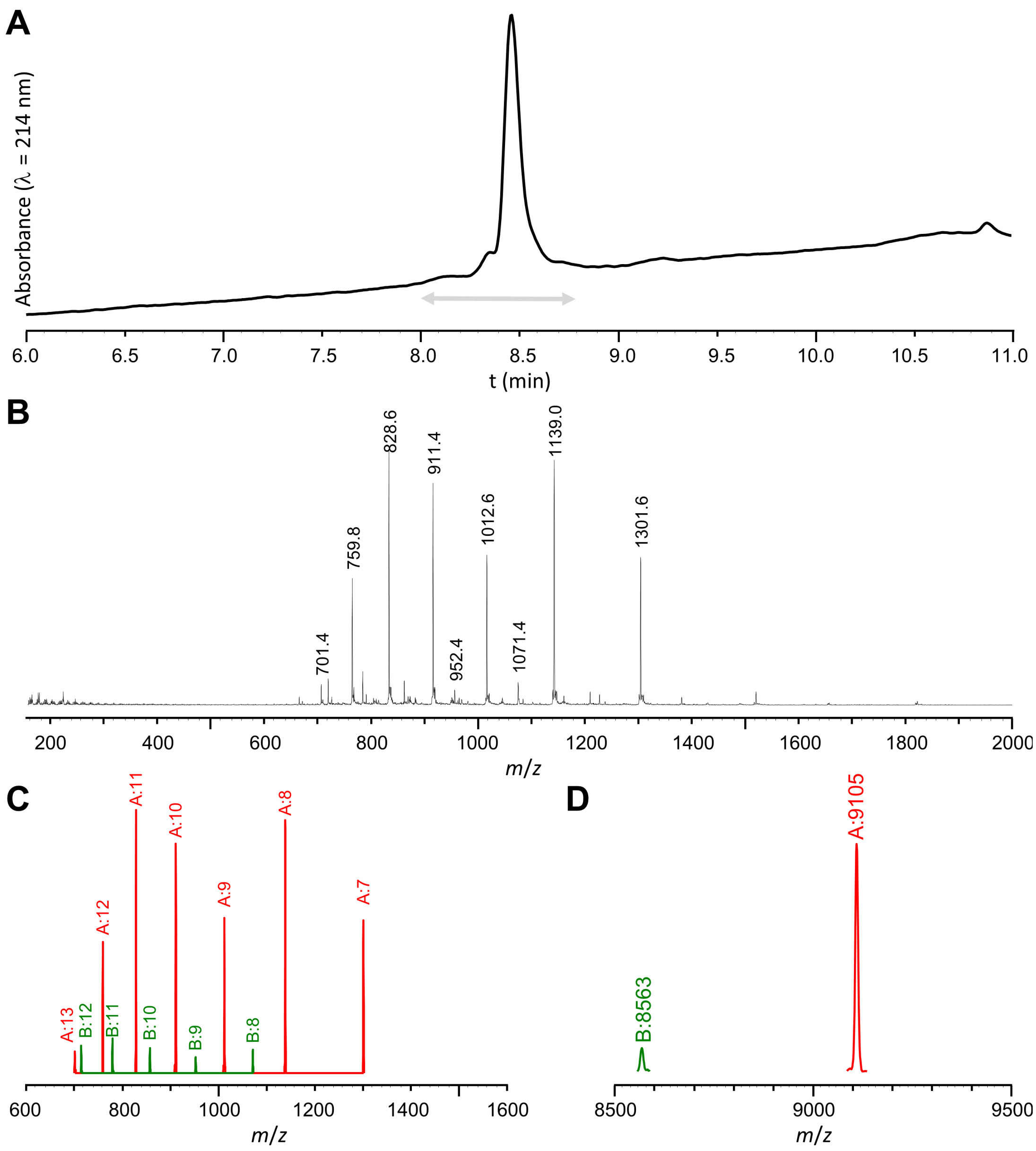

E

Compound	Relative abundance (%)	Detected average mass	Identified product, its theoretical average mass
**A**	91	9104.5 Da	Ub-ADP-ribose, 9106.0 Da
**B**	9	8563.4 Da	Ub, 8564.7 Da

Appendix Figure S3. HPLC-MS analysis of the ubiquitylation mixture performed using DTX3L-RD and ADPr.

(A) HPLC chromatogram;

(B) Experimental mass spectrum corresponding to the time window indicated as a grey arrow (sum of spectra);

(C) Deconvoluted ions set, including charge state;

(D) Deconvoluted spectrum;

(E) Identified compounds.

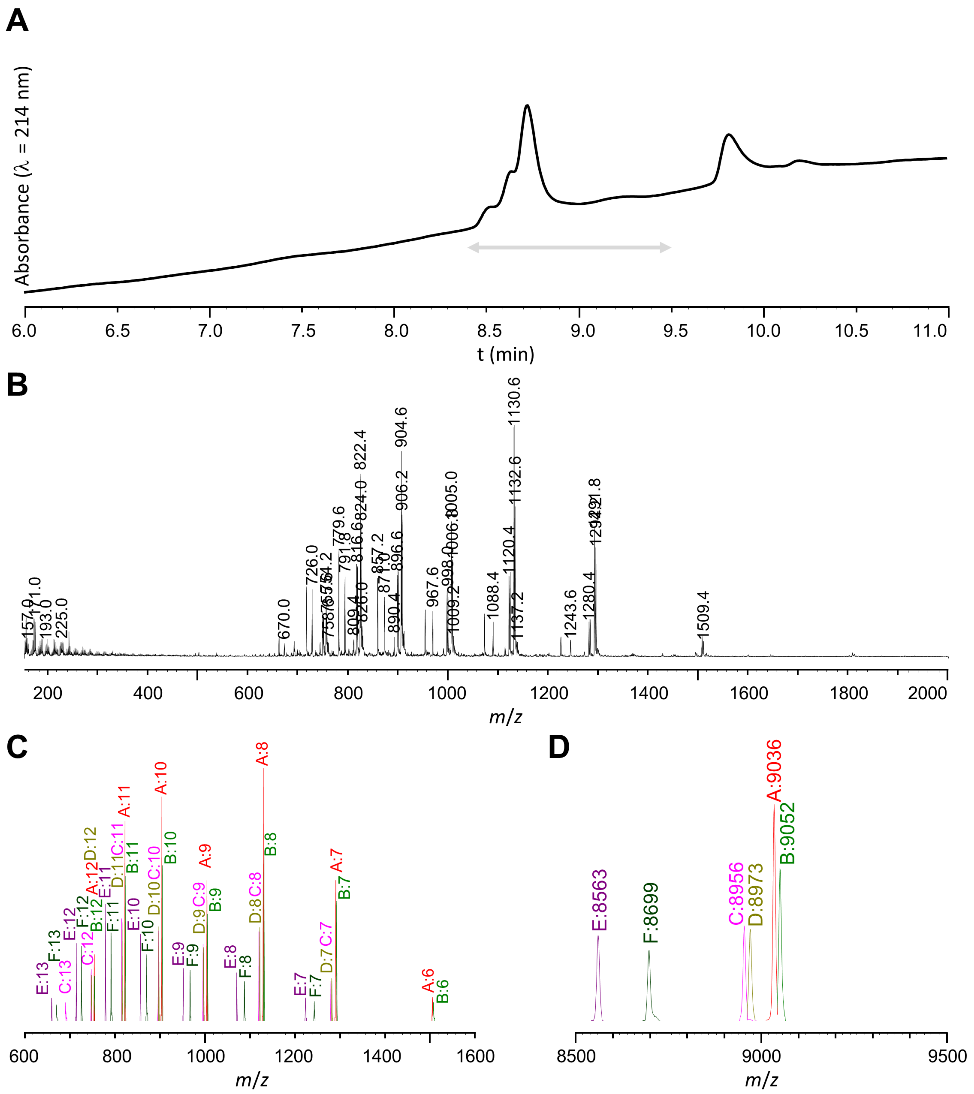

E

Compound	Relative abundance (%)	Detected average mass	Identified product, its theoretical average mass
A	31	9036.4	Ub-2’dATP, 9037.9 Da
B	21	9052.3	Ub-ATP, 9053.9 Da
C	13	8956.4	-
D	13	8972.6	Ub-ADP, 8973.9 Da
E	12	8563.2	Ub, 8564.7 Da
F	10	8699.2	Ub-DTT, 8701.1 Da

Appendix Figure S4. HPLC-MS analysis of the ubiquitylation mixture performed using DTX3L-RD and 2’ deoxy-ATP.

(A) HPLC chromatogram;

(B) Experimental mass spectrum corresponding to the time window indicated as a grey arrow (sum of spectra);

(C) Deconvoluted ions set, including charge state;

(D) Deconvoluted spectrum;

(E) Identified compounds.

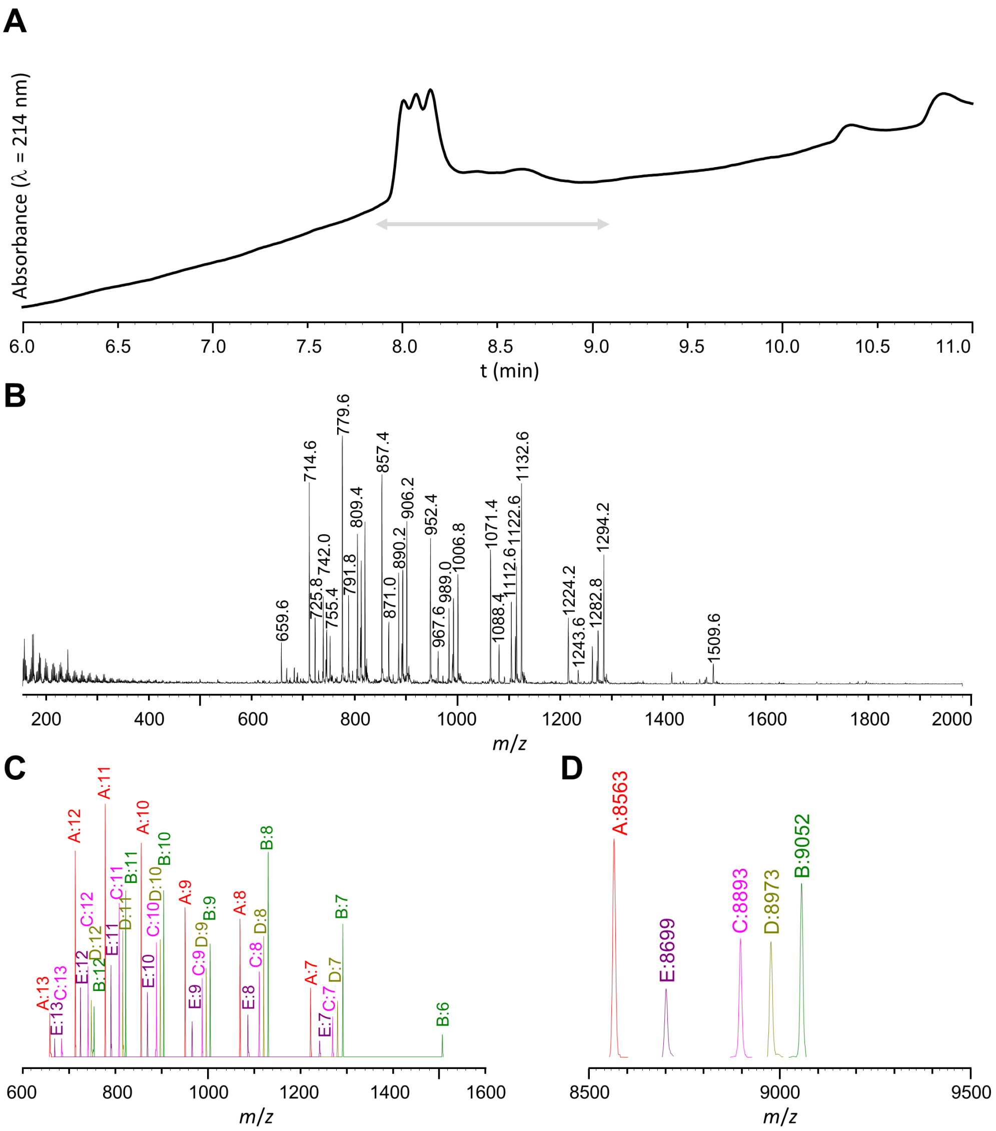

E)

Compound	Relative abundance (%)	Detected average mass	Identified product, its theoretical average mass
A	31	8563.5	Ub, 8564.7 Da
B	25	9052.3	Ub-ATP, 9053.9 Da
C	17	8892.6	Ub-AMP, 8893.9 Da
D	17	8972.5	Ub-ADP, 8973.9 Da
E	10	9699.0	Ub-DTT, 8701.1 Da

Appendix Figure S5. HPLC-MS analysis of the ubiquitylation mixture performed using DTX3L-RD and 3’ deoxy-ATP.

(A) HPLC chromatogram;

(B) Experimental mass spectrum corresponding to the time window indicated as a grey arrow (sum of spectra);

(C) Deconvoluted ions set, including charge state;

(D) Deconvoluted spectrum;

(E) Identified compounds.

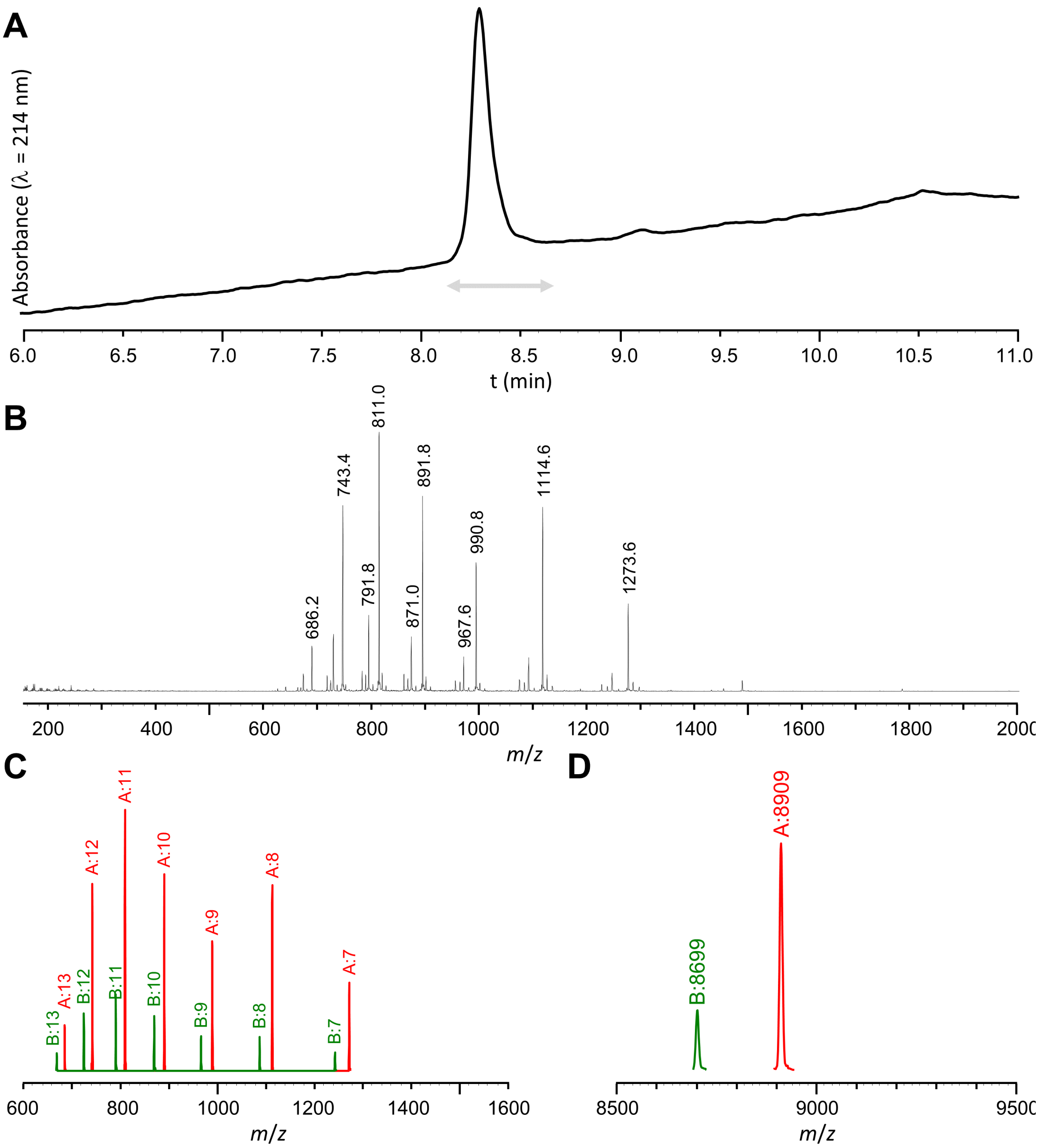

E

Compound	Relative abundance (%)	Detected average mass	Identified product, its theoretical average mass
A	79	8908.9 Da	Ub-GMP, 8909.9 Da
B	21	8699.5 Da	Ub-DTT, 8701.1 Da

Appendix Figure S6. HPLC-MS analysis of the ubiquitylation mixture performed using DTX3L-RD and GMP.

(A) HPLC chromatogram;

(B) Experimental mass spectrum corresponding to the time window indicated as a grey arrow (sum of spectra);

(C) Deconvoluted ions set, including charge state;

(D) Deconvoluted spectrum;

(E) Identified compounds.

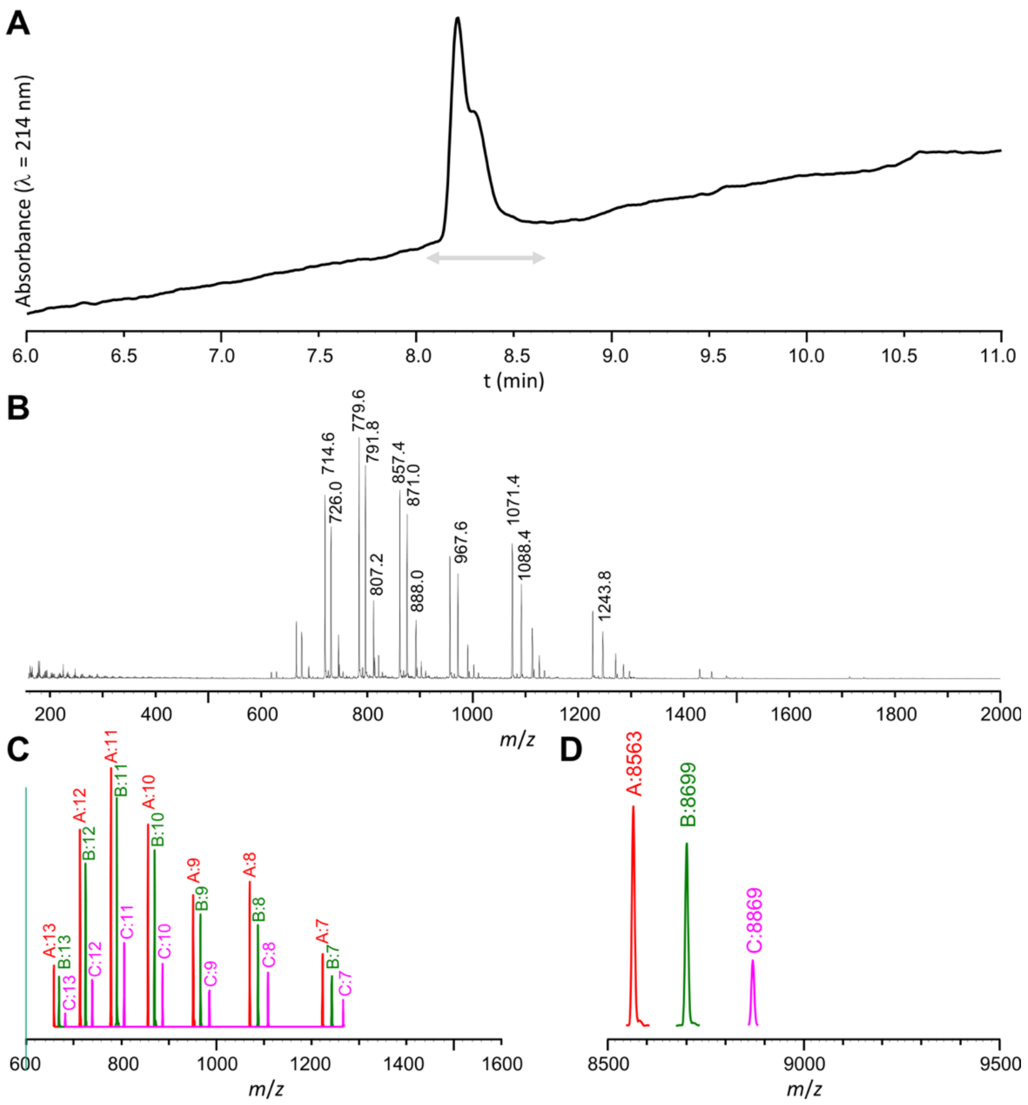

E

Compound	Relative abundance (%)	Detected average mass	Identified product, its theoretical average mass
A	47	8563.5 Da	Ub, 8564.7 Da
B	39	8699.5 Da	Ub-DTT, 8701.1 Da
C	14	8868.6 Da	Ub-CMP, 8869.9 Da

Appendix Figure S7. HPLC-MS analysis of the ubiquitylation mixture performed using DTX3L-RD and CMP.

(A) HPLC chromatogram;

(B) Experimental mass spectrum corresponding to the time window indicated as a grey arrow (sum of spectra);

(C) Deconvoluted ions set, including charge state;

(D) Deconvoluted spectrum;

(E) Identified compounds.

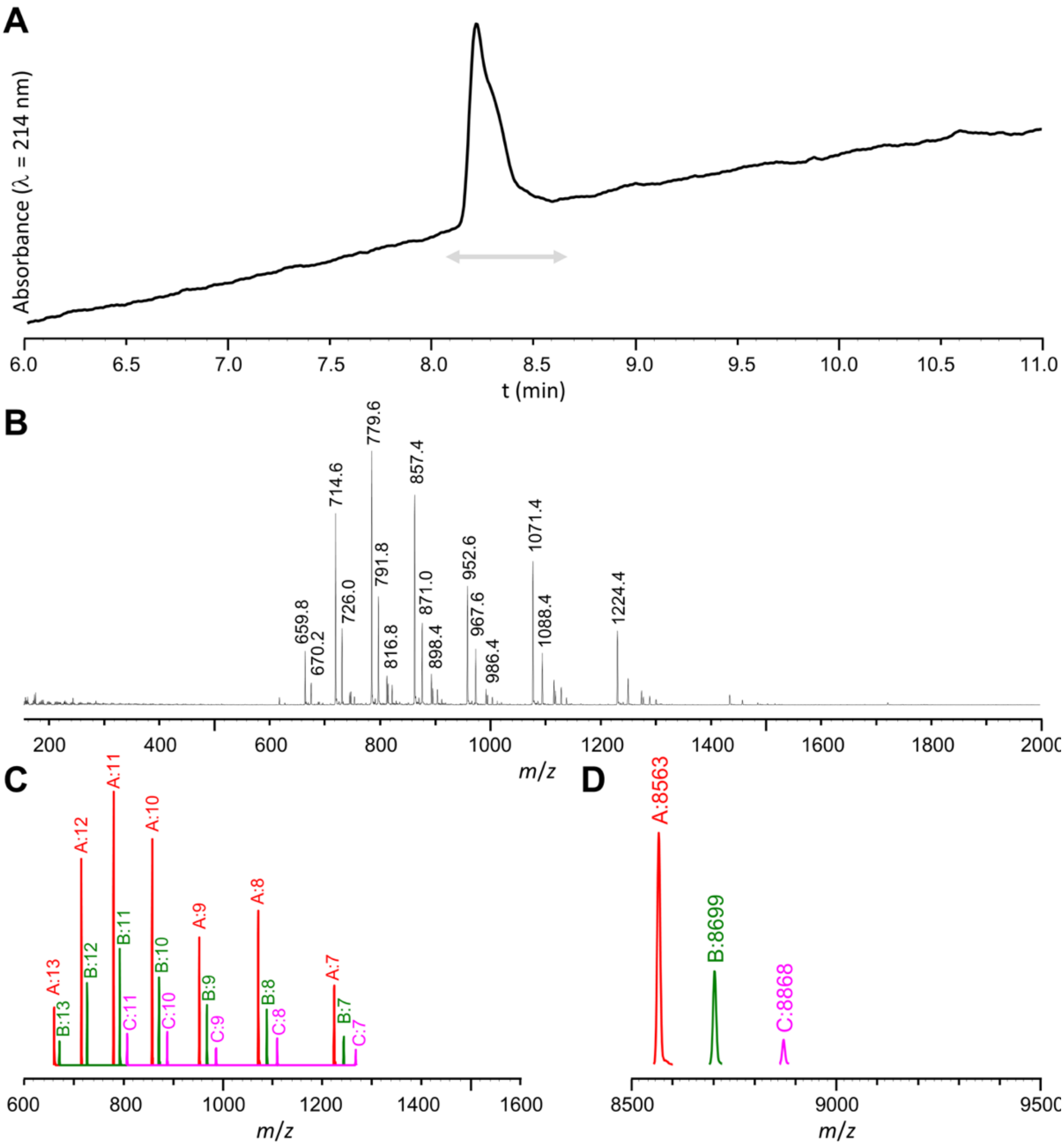

E

Compound	Relative abundance (%)	Detected average mass	Identified product, its theoretical average mass
A	66	8563.5 Da	Ub, 8564.7 Da
B	27	8699.3 Da	Ub-DTT, 8701.1 Da
C	7	8867.6 Da	Ub-TMP, 8868.9 Da

Appendix Figure S8. HPLC-MS analysis of the ubiquitylation mixture performed using DTX3L-RD and TMP.

(A) HPLC chromatogram;

(B) Experimental mass spectrum corresponding to the time window indicated as a grey arrow (sum of spectra);

(C) Deconvoluted ions set, including charge state;

(D) Deconvoluted spectrum;

(E) Identified compounds.

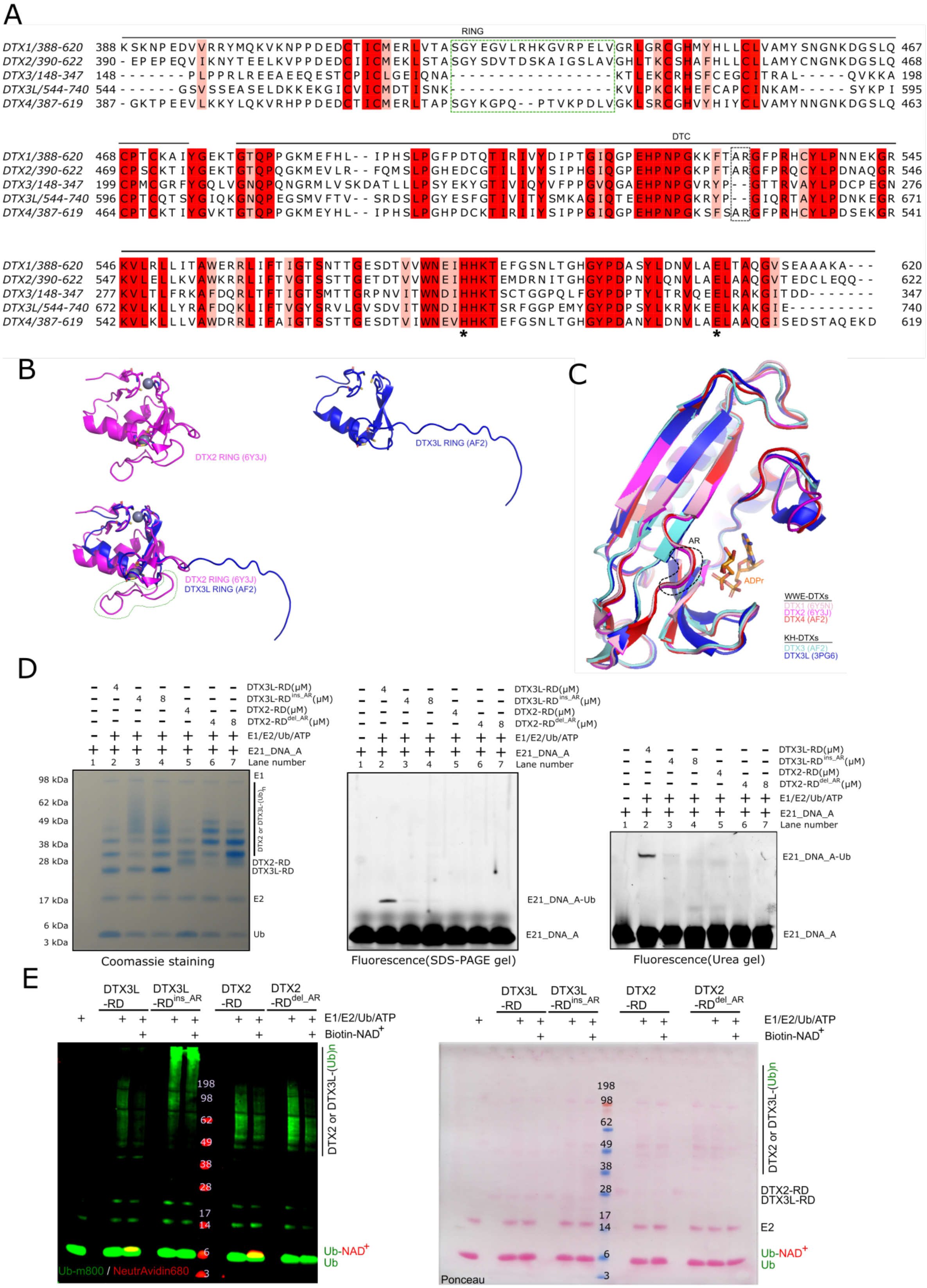

Appendix Figure S9. Differences between WWE domain-containing DELTEXes and KH domain-containing DELTEXes.

(A) Sequence alignment of the RD domains DELTEX E3s. Sequence conservation is colored in shades of red (red=identical, pink=conserved). The conserved catalytic residues in the DTC domain (DTX3L H707, E733) are indicated by asterisks. Differences in the RING domain and AR loop in DTC domain are indicated by green or black dashed box. Sequence alignment was generated using Clustal Omega(Sievers & Higgins, 2018) and Jalview(Waterhouse, Procter et al., 2009).
(B) Comparison of the RING domains of DTX2 and DTX3L. The Zn^{2+}-coordinating residues are shown in sticks, Zn^{2+} atoms are shown as grey spheres. The extension in RING domain of DTX2 is marked by green dashed line.
(C) Alignment of the DTC domains of DELTEX E3s. Extra AR residues in DTX1, DTX2, or DTX4 creates a protrusion, marked by black dashed line.
(D) Validation of the structural analysis in (A and C) using AR deleted DTX2-RD mutant or AR inserted DTX3L-RD mutant.
(E) NAD^+ ubiquitylation assay for DTX3L-RD, DTX3L-RD^{ins_AR}, DTX2-RD, and DTX2-RD^{del_AR}. Reactions were resolved using SDS-PAGE followed by western blot, and detected using antibodies for Ub (PD41) in green and NeutrAvidin680 in red.
