# Supplementary material for: Ubiquitylation of nucleic acids by DELTEX ubiquitin E3 ligase DTX3L
Source: EMBO Rep. 2024 Sep 6;25(10):11. doi: 10.1038/s44319-024-00235-1 (PMC11467253; doi:10.1038/s44319-024-00235-1)
Supplement: Supplementary file 3 — Expanded View Figures [file 44319_2024_235_MOESM3_ESM.pdf]

Expanded View Figures

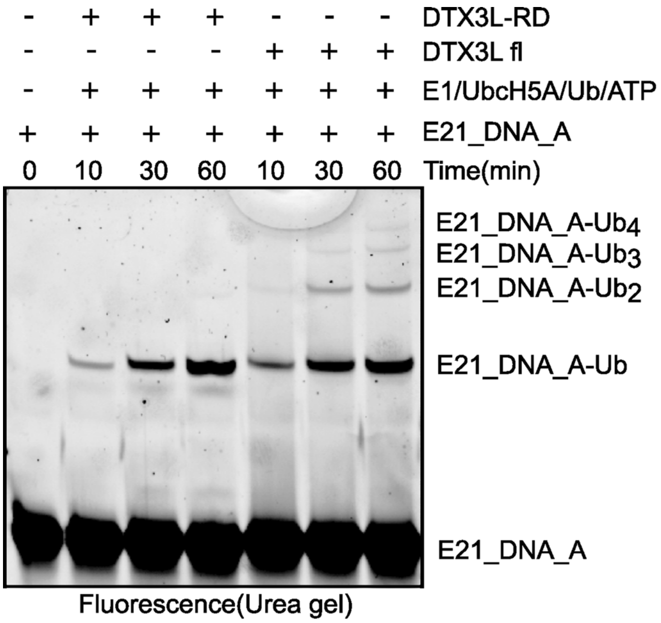

Figure EV1. DTX3L catalysed nucleic acids ubiquitylation.

E21_DNA_A was ubiquitylated by DTX3L-RD and DTX3L fl, at indicated time points. The experiment has been completed in triplicate.

 © The Author(s)

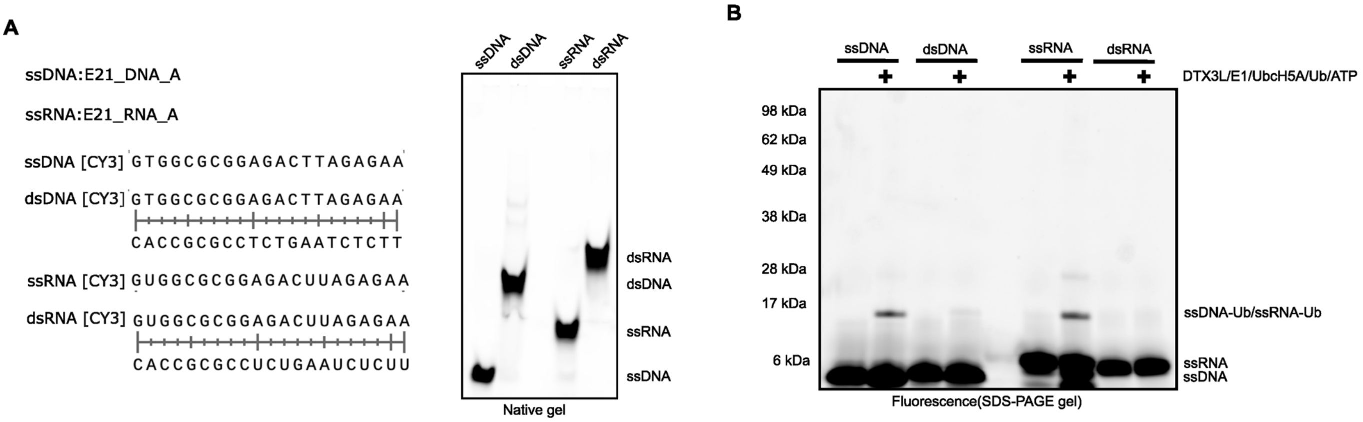

Figure EV2. DTX3L-RD is not able to ubiquitylate double-stranded nucleic acids.

(**A**) Annealed dsDNA and dsRNA were visualised on native gel. (**B**) dsDNA and dsRNA ubiquitylation assay. The experiment has been completed in triplicate.

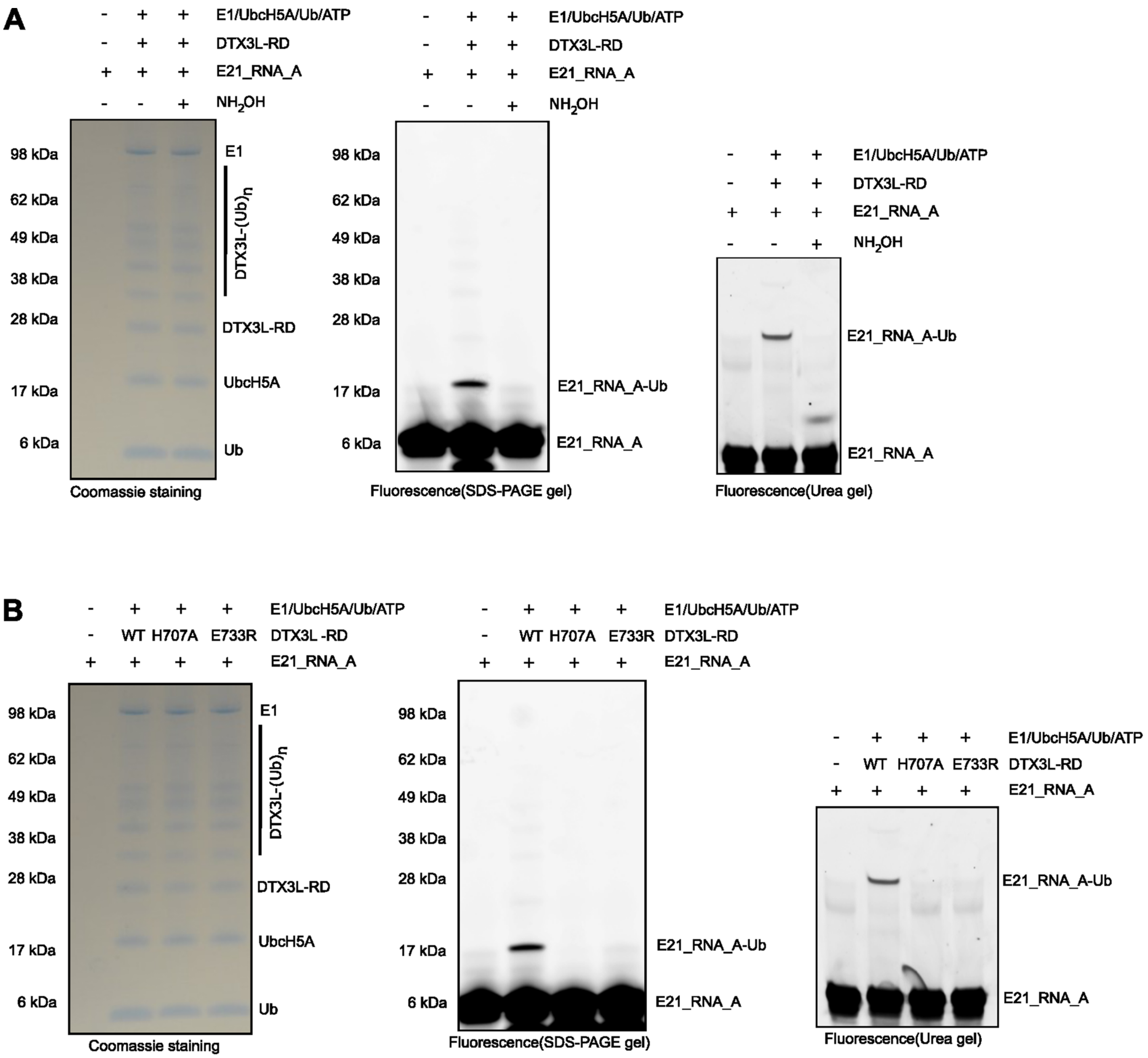

Figure EV3. DTX3L-RD attaches Ub onto the 3′ hydroxyl group of terminal adenosine in RNA.

(**A**) NH_2OH reverses DTX3L-RD-catalysed nucleic acids ubiquitylation. NH_2OH cleaves the ester bond between the carbonyl group of Gly^{76} of Ub and the 3′ hydroxyl group of the A of E21_RNA_A. (**B**) DTX3L-RD ADPr ubiquitylation inactive mutants failed to produce upshift bands that correspond to ubiquitylation of RNA, indicating that Ub is attached to 3′ hydroxyl group. Each experiment has been completed in triplicate.

 © The Author(s)

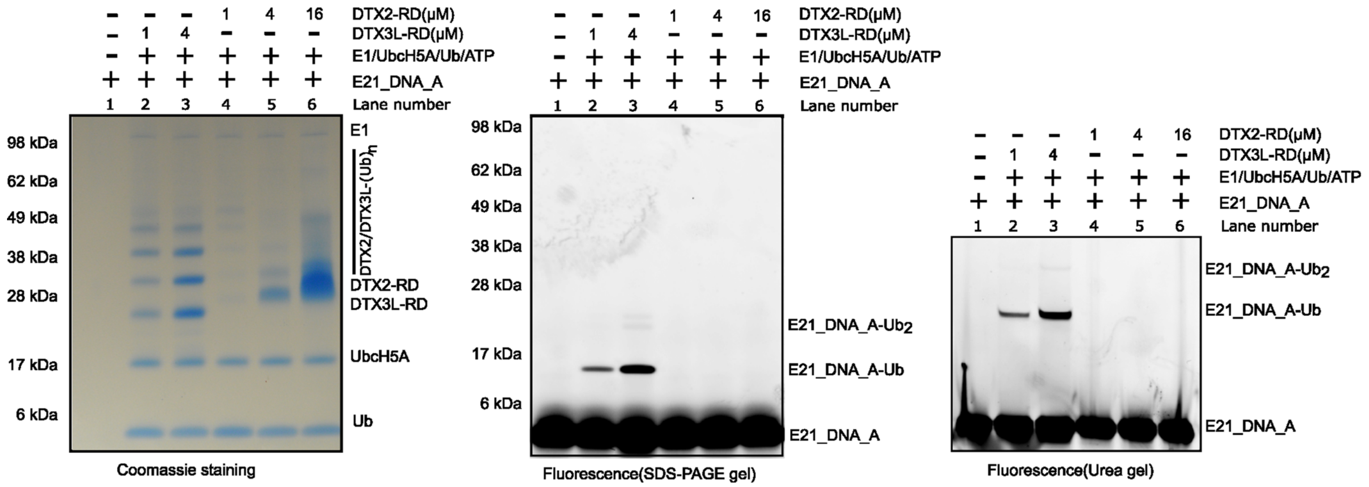

Figure EV4. DTX2-RD is not able to ubiquitylate nucleic acids.

E21_DNA_A was incubated with E1, E2 UbcH5A, ATP, Ub and increasing amount of either DTX3L-RD or DTX2-RD, then the reactions were analysed on an SDS-PAGE gel and Urea gel and visualized using the Molecular Imager PharosFX system (BioRad). The experiment has been completed in triplicate.

© The Author(s)

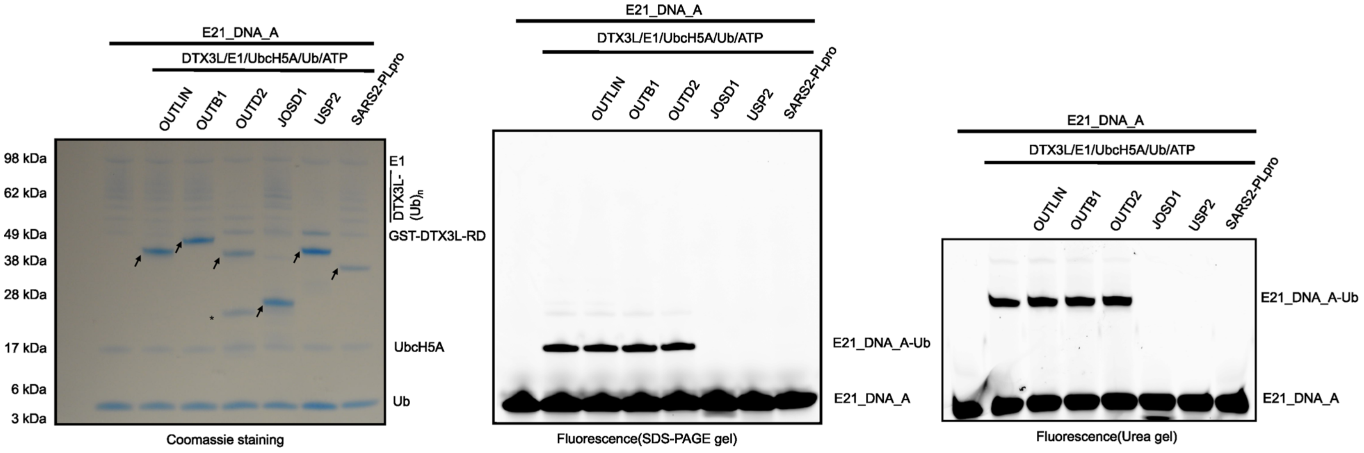

Figure EV5. DNA ubiquitylation reactions treated with a panel of DUBs.

Arrows indicate DUBs, asterisk indicates contaminant band.

© The Author(s)
